# Age-Related Associations of Foveal Structural Parameters in Healthy Adults: A Comparative Analysis of Biological and Chronological Age

**DOI:** 10.3390/vision10010016

**Published:** 2026-03-03

**Authors:** Anait S. Khalatyan, Yusef Yusef, Khadishat K. Altemirova, Liubov V. Machekhina, Alexandra A. Melnitskaya, Irina D. Strazhesko

**Affiliations:** 1 Krasnov Research Institute of Eye Diseases, 11 A, B, Rossolimo St., Moscow 119021, Russia; info@eyeacademy.ru (Y.Y.); hadishka_16@mail.ru (K.K.A.); 2 Russian Gerontology Research and Clinical Centre, Pirogov National Research Medical University, Moscow 129226, Russia; machehina_lv@rgnkc.ru (L.V.M.); melnickaya_aa@rgnkc.ru (A.A.M.); strazhesko_id@rgnkc.ru (I.D.S.)

**Keywords:** fovea, retinal aging, optical coherence tomography (OCT), biological age, PhenoAge, foveal bulge, foveal pit

## Abstract

Background: This research compared the relationship between foveal optical coherence tomography (OCT) parameters and two age measures—biological and chronological—in healthy adults. Methods: This cross-sectional study analyzed swept-source optical coherence tomography (OCT) data from 308 eyes of 154 healthy adults aged 22–89 years. Parameters assessed: foveal thickness, foveal pit depth and diameter, pit slope steepness, and the presence or absence of the foveal bulge. Biological age was calculated using the PhenoAge algorithm. Results: The core geometry of the foveal pit showed no significant dependence on either type of age (all *p* ≥ 0.66). In contrast, the foveal bulge prevalence declined significantly with age (adjusted *p* = 0.011 for chronological age, *p* = 0.005 for biological age; OR per year ≈0.95, 95% CI 0.92–0.98 for both age models). Model-predicted prevalence decreased from approximately 93% in younger adults to 60–68% in the 60–74-year-old group. Conclusion: The foveal architecture remains structurally stable throughout adulthood. The foveal bulge emerges as a sensitive qualitative marker of age-related changes. Biological age does not provide additional predictive value over chronological age for foveal structural parameters under physiological aging conditions.

## 1. Introduction

The fovea, being the central part of the macula, possesses a unique microstructure. The characteristic pit, formed by the displacement of the inner retinal layers, the maximal density of cone photoreceptors, and minimal light scattering provide optimal conditions for photopic (daylight) vision [1,2]. Individual variations in foveal pit morphology are common in the normal population [3], making an accurate understanding of its normative, non-pathological aging trajectories crucial for differential diagnosis from early-stage disease.

Optical coherence tomography (OCT) has become the standard for in vivo visualization of retinal microstructure. Aging is known to be associated with progressive thinning of the retinal nerve fiber layer, the ganglion cell complex, as well as choroidal atrophy [4]. However, data on the sensitivity of fine foveal parameters, such as pit configuration, foveal thickness, and the integrity of the photoreceptor outer segments, to age-related changes remain inconsistent.

Of particular interest is the foveal bulge—a convex configuration of the photoreceptor outer segment and ellipsoid zone complex at the center of the foveola [5]. Its presence is considered an indicator of structural photoreceptor integrity and correlates with better functional outcomes in various macular pathologies [6,7,8,9]. Despite its potential clinical significance, population data on the age-related dynamics of this feature’s prevalence are limited.

Traditionally, modeling retinal aging has been based on chronological age [10]. Recently, the concept of biological age has been proposed as a more accurate reflection of cumulative physiological wear and tear and tissue-specific processes [11]. Since photoreceptors, especially the cones of the foveola, have extremely high metabolic demands [12], their microstructure may be particularly sensitive to systemic markers of biological aging, such as oxidative stress and mitochondrial dysfunction.

One of the most validated measures of biological age is PhenoAge—an algorithm that integrates information from 9 clinical and biochemical markers along with chronological age, and has proven its prognostic value for mortality and age-associated diseases [13]. Hypothetically, PhenoAge could capture subtle changes in the metabolically active fovea earlier or more accurately than chronological age.

Rather than assuming the superiority of biological age over chronological age, this study aims to test the extent to which foveal microstructure follows systemic biological aging versus chronological time in healthy adults. Identifying parameters that deviate from chronological synchrony may provide insights into retinal resilience and vulnerability to age-related remodeling.

The aim of this study is to conduct a comparative analysis of the associations between a comprehensive set of OCT-derived parameters of the fovea and both biological (PhenoAge) and chronological age in a cohort of healthy adults.

## 2. Materials and Methods

### 2.1. Demographic Characteristics of Study Participants

This cross-sectional analysis utilized data collected as part of the RussAge research project. The sample consisted of 154 healthy Caucasian volunteers (308 eyes) aged 22 to 89 years. The study was conducted at the M.M. Krasnov Research Institute of Eye Diseases in collaboration with the Russian Gerontological Research and Clinical Center, Pirogov Russian National Research Medical University (RNRMU). The study protocol was approved by the Local Ethics Committee (protocol No. 59 from 13 September 2022). All participants provided written informed consent, and the study adhered to the tenets of the Declaration of Helsinki.

Inclusion criteria were: age ≥ 18 years, ability to provide informed consent.

Exclusion criteria were applied to establish a cohort healthy in terms of retinal and systemic status:

- Any current or past ophthalmic pathology: refractive errors exceeding ±3.0 diopters, best-corrected visual acuity of 0.8 decimal (Snellen equivalent 20/25) or worse, cataract, glaucoma, uveitis, diabetic retinopathy, age-related macular degeneration, retinal vascular occlusion, optic neuropathy.
- History of intraocular surgery (except for uncomplicated cataract surgery performed more than 6 months prior).
- Systemic diseases potentially affecting the retina or metabolism: diabetes mellitus of any type, autoimmune diseases (e.g., rheumatoid arthritis, systemic lupus erythematosus), active oncological disease or a history of chemotherapy, diagnosed dementia (Mini-Mental State Examination score < 24) or severe psychiatric disorders.
- Poor-quality OCT scans. Image quality was assessed using the proprietary Topcon Image Quality index (scale 0–100), which evaluates the signal-to-noise ratio and the clarity of the retinal layers. Only scans with a quality score of 90 or higher were included in the analysis to ensure reliable boundary detection. Out of the initial 312 eyes scanned, 4 eyes from 2 participants were excluded due to poor image quality (score < 90), resulting in a final dataset of 154 participants (308 eyes).

### 2.2. OCT Imaging and Image Analysis

All participants underwent a comprehensive ophthalmic examination, including visometry, autorefractometry, and slit-lamp biomicroscopy of the anterior segment and fundus.

Scanning Protocol: Macular imaging was performed by a single operator using the same device, the swept-source optical coherence tomography (SS-OCT) with a high-definition raster (‘HD Raster’) scan protocol (Triton DRI OCT, Topcon Corporation, Tokyo, Japan). All scans were performed through undilated pupils under mesopic lighting conditions using the internal fixation target.

Image Analysis and Segmentation: Automatic segmentation of all retinal layers was performed using the device’s built-in software (version: IMAGEnet 6 Version 1.32.18683; Topcon Corporation, Tokyo, Japan). The quality of segmentation and centration for each scan was manually verified by two independent graders masked to participant age. In cases of incorrect automatic segmentation, manual boundary correction was performed. Manual boundary correction was required for 7 scans (2.27% of the total sample). These corrections were performed by two independent, masked graders, who were blinded to the subjects’ chronological and biological ages. Discrepancies were resolved by consensus. The reliability of the semi-automated segmentation has been previously validated against manual tracing performed by five retinal experts on a subset of 30 scans, yielding an Intraclass Correlation Coefficient (ICC) > 0.95 for all parameters (see Appendix A Table A1 for details).

**Figure 1:**
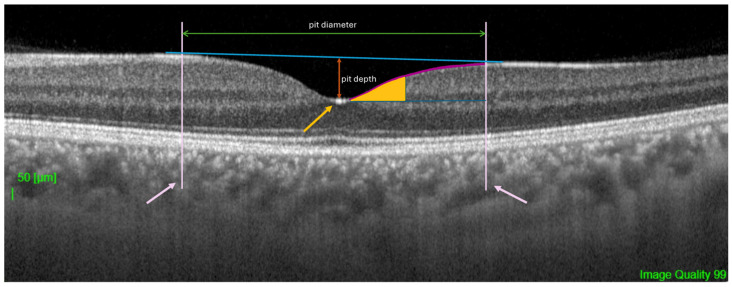
Definition of foveal pit geometric parameters on a horizontal swept-source OCT B-scan. The internal limiting membrane (ILM) profile is shown. The pit edges are indicated by pink arrows and correspond to the locations where the first derivative of the ILM profile reaches 50% of the maximum slope on either side of the foveal center; the same locations are marked by the vertical pink lines. The pit floor, defined as the point of minimum ILM thickness, is indicated by a yellow arrow. The shaded area represents the pit walls used for mean slope calculation. The reference plane (blue line above the ILM) connects the pit edges and serves as the baseline for pit depth measurement. Pit depth is illustrated by the red vertical arrow and is measured as the vertical distance between the pit floor and the reference plane. The pit diameter is defined as the horizontal distance between the pit edges. Scale bar: 50 µm.

**Figure 2:**
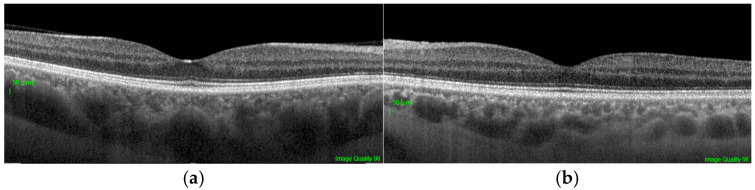
Representative swept-source OCT B-scans illustrating qualitative assessment of the foveal bulge. (**a**) Presence of the foveal bulge in healthy subjects, visible as a convexity of the ellipsoid zone or ellipsoid zone–outer photoreceptor segment complex at the foveal center; (**b**) Absence of the foveal bulge, characterized by a flattened ellipsoid zone profile at the foveal center. Scale bar: 50 µm.

**Figure A1:**
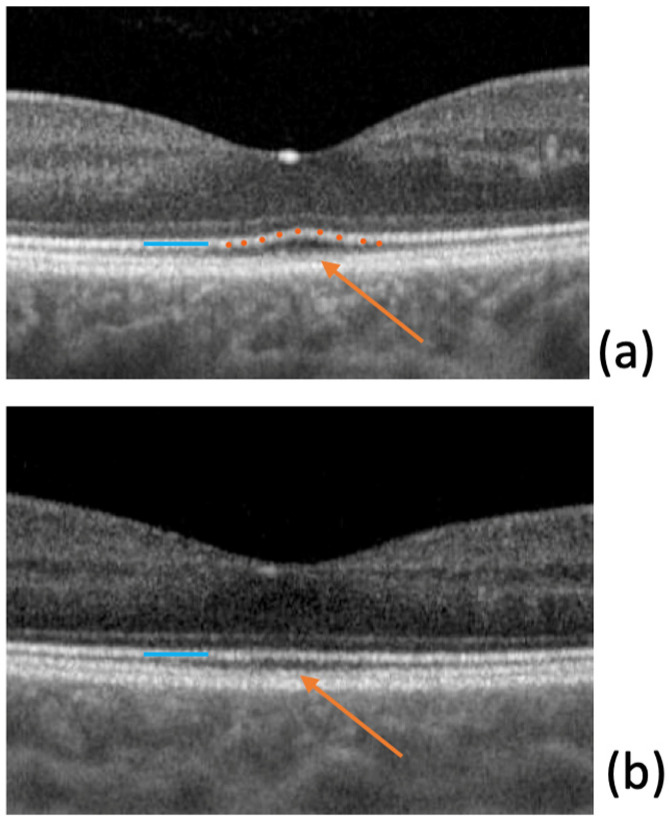
Representative annotated swept-source OCT B-scans illustrating the qualitative grading of the foveal bulge. Orange dotted line depicts the local elevated profile of the ellipsoid zone at the foveal center, while blue lines indicate the reference ellipsoid zone level used for visual assessment. Arrows highlight the region of interest used for qualitative grading. (**a**) Presence of the foveal bulge; (**b**) Absence of the foveal bulge.

The following quantitative and qualitative parameters were derived from the scan data, defined relative to the anatomical center of the fovea (Figure 1):

- Central Foveal Thickness (CFT): The average retinal thickness within the central 1 mm diameter circle.
- Foveal Pit Depth: The vertical distance between the pit floor (the point of minimum thickness of the internal limiting membrane, ILM) and a reference plane connecting its edges. The edges were defined as the points where the first derivative of the ILM profile reached 50% of the maximum slope value. This relative, percentage-based threshold was selected to balance sensitivity to changes in pit slope with robustness to local noise in the ILM contour. Similar derivative- and model-based approaches have been previously applied in quantitative analyses of foveal morphology [1,14]. To evaluate the robustness of this edge definition, a sensitivity analysis was performed using alternative derivative thresholds (40% and 60% of the maximum slope). The resulting measurements of pit depth and diameter showed high agreement with those obtained using the primary 50% threshold, indicating that the geometric parameters were not materially dependent on the specific threshold choice. Details of this analysis are provided in Appendix A (Table A2).
- Foveal Pit Diameter: The horizontal distance between the two edges defined above.
- Mean Foveal Pit Slope: The mean absolute angle of the pit walls between its edge and floor as described in previous studies [1,14].
- Presence of Foveal Bulge: This was assessed visually by two independent, masked graders on magnified B-scan tomograms (Figure 2a,b). The bulge was defined as a visible convexity (towards the vitreous) of the ellipsoid zone (EZ) line or the EZ-outer photoreceptor segment complex. Inter-rater reliability for the total sample (308 eyes) was excellent, with an initial agreement of 98.1% (6 out of 308 scans) and a Cohen’s kappa coefficient of 0.96. Discrepancies were resolved through consensus with a third senior masked expert. Representative annotated examples illustrating the qualitative grading criteria are provided in Appendix A (Figure A1).

### 2.3. Biological Age Assessment (PhenoAge)

The biological age for each participant was calculated using the PhenoAge (phenotypic age) algorithm, as described by Levine et al. (2018) [13]. The following nine clinical-laboratory biomarkers, measured as part of a general clinical check-up in a single certified laboratory, were included in the analysis: albumin, creatinine, glucose, C-reactive protein, lymphocyte percentage, mean red cell volume, red cell distribution width, alkaline phosphatase, and leukocyte count. Chronological age was also included in the formula. The calculation was performed using the published regression coefficients. The resulting PhenoAge value is expressed in years and can be either lower or higher than the chronological age, reflecting decelerated or accelerated biological aging, respectively.

To ensure the temporal consistency of the data, blood draws were performed within 7 days before the OCT imaging. To minimize the impact of transient fluctuations due to acute inflammatory states, participants were screened for symptoms of acute infection (e.g., fever, respiratory symptoms) at the time of examination. Furthermore, individuals with a C-reactive protein (CRP) level exceeding 10 mg/L were excluded from the primary analysis, as such levels typically indicate an acute phase response rather than chronic systemic inflammation.

### 2.4. Statistical Analysis

#### 2.4.1. Data Description and Group Comparison

The sample size (*n* = 154 participants) was determined retrospectively based on data availability. Post hoc analysis confirmed adequate power to detect small-to-medium effects (minimum detectable R^2^ ≈ 0.049 in linear mixed models at 80% power and α = 0.05).

Quantitative variables are presented as medians and interquartile ranges: Me [IQR]. The Wilcoxon signed-rank test (W) was used to compare parameters between the right and left eyes. Categorical variables are described with frequency and 95% confidence intervals (CI). Differences between age groups were assessed using the McNemar’s test.

#### 2.4.2. Modeling Age-Related Variables

Age-related associations for retinal parameters were analyzed separately for chronological and biological age using generalized additive mixed models (GAMMs). All models included a random intercept per participant to account for the paired nature of the data (right and left eyes) and inter-individual variability. Continuous parameters were modeled with a Gaussian family (identity link), while the binary presence/absence of the foveal bulge was modeled with a binomial family (logit link).

For each parameter and age type, three candidate models were compared:

1. Constant model (intercept-only): no age dependence.
2. Linear model: monotonic age dependence (fixed linear age term).
3. Non-linear model: generalized additive model (GAM) with thin-plate regression spline on age; basis dimensions k = 5, 10, 15, and 20 tested, selecting the best by Akaike information criterion (AIC)).

The best-fitting model was selected based on the lowest Akaike information criterion (AIC, more details in Table A3). Population-level predictions (excluding random effects) were generated for ages 17–92 years in 1-year increments, with 95% CIs for the mean predicted value and 95% prediction intervals (PIs) incorporating residual and random-effect variance (where applicable). For clinical interpretation, predictions were averaged within four age groups (17–44, 45–59, 60–74, and 75–92 years).

The statistical significance of linear age associations was consistently assessed using Wald test *p*-values for the age coefficient from the linear mixed model (providing a conservative test of fixed age effects after accounting for inter-individual variability). *p*-values were adjusted for multiple comparisons using the Benjamini–Hochberg procedure across all parameters and both age types.

Full model diagnostics for all generalized additive (mixed) models are provided in Appendix A (Table A4).

#### 2.4.3. Correlation Analysis

For parameters with a linear age-related relationship, the Spearman correlation coefficient (rs) was additionally calculated to assess the strength of the relationship. Benjamini–Hochberg correction was applied to control for multiple comparisons.

Effects were considered statistically significant at *p* < 0.05. *p* values in the range of 0.05–0.1 were interpreted as indicating a tendency toward the presence of an effect. Statistical analyses were performed using the R programming language (version 4.4.2; R Development Core Team, 2012; Vienna, Austria).

## 3. Results

Data from 154 participants were included in the final analysis. Demographic characteristics are presented in Table 1. The age distribution was uniform, covering young, middle-aged, and older adults. The mean chronological age was 49.5 years (SD ± 15.8), and the mean biological age (PhenoAge) was 45.9 years (SD ± 13.2). A strong correlation between the two age metrics (r = 0.92, *p* < 0.001) confirmed overall consistency, yet the observed scatter (PhenoAgeAccel, the difference between PhenoAge and chronological age) indicates individual variations in the rate of aging within the participant sample.

No statistically significant differences between the right and left eye were found for any of the key structural foveolar parameters (*p* > 0.05 for all comparisons using the Wilcoxon test) (Table 2). Although no statistically significant differences were found between right and left eyes for key foveolar parameters (Table 2), all subsequent modeling incorporated data from both eyes using generalized additive mixed models with a random intercept per participant to appropriately account for within-subject correlation.

**Figure A2:**
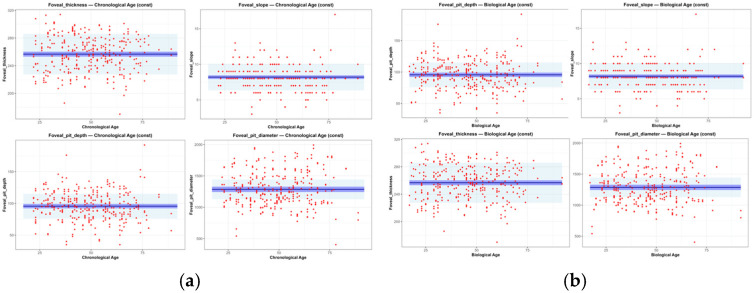
Age-related trajectories of foveal parameters based on best-fitting mixed models (constant model). Points represent individual observations; solid line—model-predicted mean; dark blue area—95% confidence interval for the mean; light blue area—95% prediction interval. (**a**) Chronological age model; (**b**) Biological age model.

Analysis using constant, linear, and nonlinear (GAM) models showed that the primary parameters describing the geometry of the foveal pit had no significant age-related dependence (Table 3 and Table 4). For all four parameters (thickness, depth, diameter, slope) and for both age measures (chronological and biological), the optimal model based on the corrected Akaike Information Criterion (AICc) was the constant model (*p* > 0.1 for all linear and nonlinear terms, Figure A2a,b). This indicates the structural stability of the core foveal architecture throughout adulthood in the studied sample of healthy participants.

As shown in Table 3 and Table 4, predicted values and confidence intervals for continuous foveal pit parameters were nearly identical for chronological age and biological age models. Neither the linear nor the non-linear (GAM) age term significantly improved model fit. Moreover, the effective degrees of freedom (edf) for the age smooth term in the candidate GAM models were close to 1 (ranging from 1.00 to 1.25 across parameters and age types), indicating that any potential non-linearity was negligible. The random intercept for participant explained a substantial proportion of the residual variance (SD Patient), highlighting considerable inter-individual variability in foveal morphology independent of age.

In contrast to the geometric parameters, the prevalence of the foveal bulge demonstrated a pronounced negative age-related dependence (Table 5). Logistic Regression: Each year of increase in chronological age was associated with a 5% reduction in the odds of the bulge being present (OR = 0.949, 95% CI: 0.918–0.981, *p* = 0.011). A similar, but slightly stronger, association was observed for the biological age PhenoAge (OR = 0.946, 95% CI: 0.916–0.976, *p* = 0.005).

Model-predicted prevalence of the foveal bulge decreased markedly with age. For chronological age, the predicted probability declined from 93% (95% CI: 29–100%) in the 17–44-year group to 68% (95% CI: 5–99%) in the 60–74-year group. A similar pattern was observed for PhenoAge, with predicted prevalence decreasing from 92% (95% CI: 26–100%) in the youngest group to 60% (95% CI: 4–98%) in the 60–74-year group. The model suggested a further decline in the oldest age group (75–92 years), but estimates were associated with very wide confidence intervals due to the smaller number of participants in this category.

The predicted age-related trajectories for all parameters, derived from models using chronological age and PhenoAge, were practically identical (see overlapping confidence intervals in Table 3). The correlation between the predicted parameter values for the two age types exceeded 0.99 for all continuous variables. For the foveal bulge, the models showed comparable performance, with slight differences in AIC (Table A3). Thus, in this sample, biological age (PhenoAge) did not reveal fundamentally different or stronger associations with the structural parameters of the foveola compared to chronological age.

## 4. Discussion

The present study provides a nuanced view of retinal aging by demonstrating that, in healthy adults, most geometric features of the foveal pit remain remarkably stable and closely synchronized with chronological time. Importantly, the absence of added predictive value of biological age does not diminish the relevance of the findings but instead highlights the robustness of foveal architecture under physiological aging conditions.

The main results revealed two divergent patterns: structural conservation of the main geometric characteristics of the foveal pit and pronounced age-related sensitivity of a qualitative feature such as the foveal bulge. At the same time, contrary to the initial hypothesis, biological age assessed by the PhenoAge algorithm did not provide additional explanatory value beyond chronological age in predicting these changes. Although the association with foveal bulge reached a lower *p*-value for biological age, effect sizes, confidence intervals, and model-predicted trajectories were virtually identical between the two age metrics.

With respect to age-related changes in retinal thickness, previous studies have reported heterogeneous findings. Some authors have described a significant age-associated thinning of the total retina and nerve fiber layer [15], whereas others found no relationship between age and macular thickness in healthy eyes [16]. Regional analyses have further suggested that age-related thinning predominantly affects parafoveal and peripheral regions, while foveal thickness remains relatively preserved [17].

In another study, analysis was intentionally restricted to the foveal pit region, and no significant age-related dependence was detected for central foveal thickness or other geometric parameters [3,18]. These findings support the notion that the core architecture of the foveal pit demonstrates substantial structural resilience to physiological aging, in contrast to more peripheral retinal regions that may exhibit greater age sensitivity.

The analysis data showed that foveal thickness, foveal pit depth and diameter, and the steepness of its slope remain unchanged throughout adult life (from 22 to 89 years). This stability in a healthy cohort supports the concept that the basic architecture of the fovea, formed during development, is a highly resilient structure, minimally susceptible to involutional changes. Importantly, in our study this stability remained unchanged after accounting for inter-eye correlation using mixed-effects models, supporting the robustness of these findings. It can be assumed that such stability serves as a mechanism for maintaining the optical quality of the central pit as the “visual window,” ensuring a constant retinal image at the photoreceptor level even against the backdrop of age-related changes occurring in other layers. Thus, the revealed robustness of the main geometric parameters allows them to be considered reliable, age-independent references in clinical practice. The predictive intervals illustrate the expected range of normal foveal structural variability at a given age in healthy individuals. In clinical practice, values falling outside these intervals may prompt closer follow-up or additional evaluation, particularly for qualitative features such as the foveal bulge. However, these intervals should be interpreted as age-adjusted reference ranges rather than strict diagnostic cut-offs. These conclusions are further supported by generalized additive mixed modeling, which consistently selected intercept-only models and demonstrated negligible non-linearity across all parameters.

The key finding of this study was the clear, monotonic decline in the prevalence of the foveal bulge with age, confirmed by both age models. The 5% reduction in the odds of its presence per year (OR ~0.95) translates into an almost twofold drop in prevalence between the youngest (<45 years) and oldest (>75 years) groups. Studies in healthy adults have shown that the prevalence and height of the foveal bulge decrease with age, consistent with age-related changes in outer retinal structure [19]. This is particularly noteworthy as the presence of this structure is a known predictor of superior visual outcomes in various macular disorders [5,8,20]. The progressive loss of the bulge during healthy aging may therefore indicate a decline in the fovea’s functional reserve, potentially representing a bridge between physiological senescence and the early stages of age-related macular pathologies. Importantly, our findings establish the first normative age-related trajectory for foveal bulge prevalence in a healthy population, supporting its use as an age-adjusted qualitative biomarker of outer retinal aging in clinical OCT interpretation.

Since the foveal bulge morphologically represents a convexity formed by the cone outer segments and the ellipsoid zone, its disappearance may be associated with subtle remodeling processes in the outer retinal layers. While the disappearance of the foveal bulge with age was clearly observed in our cohort, the underlying cellular mechanisms remain to be fully elucidated. We hypothesize that this structural change may reflect a gradual shortening of the cone outer segments (COS) or a reduction in the density of foveal photoreceptors. Such alterations could potentially be linked to age-related declines in the metabolic efficiency of the retinal pigment epithelium (RPE), which plays a key role in COS phagocytosis and maintenance [21]. In line with this hypothesis, previous studies have proposed that senescence involves not only COS shortening but also altered cone orientation, changes in foveolar cone density, and functional alterations at the photoreceptor–RPE interface [22]. However, these mechanisms remain speculative based on current OCT technology. Our findings align with some histological reports on the reduction in foveal cone lengths with age, but they also highlight the need for further validation [23,24]. Future studies employing multimodal imaging, such as Adaptive Optics OCT (AO-OCT) or OCT-Angiography, are required to visualize individual photoreceptor mosaics and confirm whether these structural changes indeed correspond to specific cellular loss or morphological shortening [25,26,27].

Although biological age is conceptually designed to capture cumulative physiological stress, several factors may explain why PhenoAge did not outperform chronological age in predicting foveal structural parameters in this healthy cohort.

First, PhenoAge incorporates chronological age as an integral component of its algorithm, which inherently limits its independence as a predictor. In populations with low systemic disease burden, such as the present cohort of carefully screened healthy volunteers, biological and chronological aging processes may remain highly synchronized.

Second, the neurosensory retina, particularly the fovea, may follow a tightly regulated aging trajectory that is less susceptible to systemic metabolic variation in the absence of overt pathology [23]. This interpretation is supported by the near-identical age-related trajectories observed for both age metrics and the extremely high correlation between model predictions.

Finally, biological age biomarkers such as PhenoAge may demonstrate greater discriminative power in populations with accelerated or heterogeneous aging, including individuals with metabolic, cardiovascular, or neurodegenerative diseases. Thus, the lack of added predictive value of PhenoAge in the present study should not be interpreted as a limitation of the concept of biological aging itself, but rather as evidence of structural resilience and temporal synchrony of foveolar aging in healthy adults.

Our findings contrast with recent studies on “retinal age” derived from fundus photography. While deep learning models can predict systemic biological age from retinal vasculature and optic disc features, our study suggests that the foveal microstructure itself is structurally resilient and does not mirror these systemic shifts [28,29,30]. This dissociates the “neurosensory” aging of the fovea from the “vascular” aging captured by retinal clocks.

## 5. Limitations

The study limitations are associated with several factors. The cross-sectional design allows for the identification of age-related associations but does not permit the establishment of true longitudinal “dynamics” or causal relationships between systemic biological aging and retinal remodeling. The small size of the oldest subgroup (>75 years), especially in the PhenoAge analysis, makes estimates for this age range less precise. The homogeneity of the sample (healthy volunteers) limits the generalizability of the results to populations with comorbid conditions, where the discrepancy between biological and chronological age may be significant. In addition, ocular axial length was not explicitly accounted for, which is known to influence OCT-based measurements due to optical magnification effects. However, the inclusion of only participants without high myopia or hypermetropia likely limited the impact of axial length variability on the reported geometric parameters. Lifestyle factors such as smoking status, BMI, and systemic hypertension were not included as covariates in our models. While the PhenoAge algorithm partially captures the physiological consequences of these factors through systemic biomarkers (e.g., C-reactive protein and glucose), future studies should incorporate these variables directly to isolate their specific impact on foveal microstructure.

## 6. Conclusions

This study demonstrates that, in healthy adults, the basic geometric parameters of the foveal pit remain structurally stable throughout adulthood, supporting their reliability as age-independent anatomical references. In contrast, the foveal bulge emerged as a highly sensitive and clinically relevant feature, whose prevalence progressively declines with age. Importantly, biological age estimated by PhenoAge did not show greater predictive power than chronological age for any of the analyzed OCT parameters, indicating a high degree of synchrony between foveal structural aging and calendar time under physiological conditions. Clinically, these findings highlight the importance of accounting for patient age when interpreting the presence or absence of the foveal bulge to distinguish normal aging from early pathological changes. Future studies should focus on longitudinal validation of these trajectories, functional correlates of foveal bulge loss, and their behavior in populations with accelerated biological aging or systemic disease, where discrepancies between biological and chronological age may have greater prognostic relevance.

## Figures and Tables

**Table 1:** Demographic and general characteristics of the study participants (*n* = 154).

Parameter	Mean
Age (years), average ± SD (range)	49.5 ± 15.8 (22–89)
Biological age, PhenoAge (years), average ± SD	45.9 ± 13.2
PhenoAgeAccel (years), average ± SD	−3.6 ± 7.8
Gender, *n* (%)	
Women	87 (56.5%)
Men	67 (43.5%)
Spherical equivalent (D), median [IQR]	−0.25 [−0.75, +0.50]

**Table 2:** Comparison of OCT of the foveal parameters of the right and left eyes (*n* = 154 pairs). The data are presented as the median.

Parameter	Right Eye	Left Eye	*p*-Value *
Foveal thickness (microns)	257.5 [241.0, 274.8]	256.5 [240.0, 275.8]	0.78
Foveal pit depth (microns)	95.0 [81.0, 109.0]	94.5 [81.0, 112.0]	0.31
Foveal pit diameter (microns)	1252 [1089, 1455]	1262 [1100, 1475]	0.07
Foveal slope steepness (°)	8.0 [7.0, 9.0]	8.0 [7.0, 9.0]	0.78
Presence of foveal bulge, *n* (%)	105 (68.2%)	106 (68.8%)	0.92

* Paired Wilcoxon test for quantitative parameters; McNemar’s test for the qualitative parameter.

**Table 3:** Results of modeling the age trends of OCT parameters of the fovea (chronological age).

Parameter	*p*-Value (Linear Age Term) ^1^	Predicted Value (Fit) [95% CI]	95% Prognostic Interval (PI)
Foveal thickness (microns)	0.77	251.0 [232.9, 269.2]	199.0–303.0
Foveal pit depth (microns)	0.66	100.5 [87.6, 113.3]	53.9–147.0
Foveal pit diameter (microns)	0.77	1346.9 [1242.2, 1451.5]	801.0–1892.8
Foveal slope steepness (°)	0.66	8.0 [6.9, 9.2]	4.4–11.6

^1^ Adjusted *p*-value (Benjamini–Hochberg) from Wald test on linear age term in mixed model (Gaussian family for continuous parameters).

**Table 4:** Results of modeling the age trends of OCT parameters of the fovea (biological age).

Parameter	*p*-Value (Linear Age Term) ^1^	Predicted Value (Fit) [95% CI]	95% Prognostic Interval (PI)
Foveal thickness (microns)	0.77	251.0 [232.9, 269.2]	199.0–303.0
Foveal pit depth (microns)	0.66	100.5 [87.6, 113.3]	53.9–147.0
Foveal pit diameter (microns)	0.77	1346.9 [1242.2, 1451.5]	801.0–1892.8
Foveal slope steepness (°)	0.66	8.0 [6.9, 9.2]	4.4–11.6

^1^ Adjusted *p*-value (Benjamini–Hochberg) from Wald test on linear age term in mixed model (Gaussian family for continuous parameters). Note: Model estimates for biological age were numerically identical to those obtained for chronological age, reflecting the absence of additional explanatory value of PhenoAge in this cohort.

**Table 5:** Results of modeling the age trends of the foveal bulge.

Parameter	Type of Age	*p*-Value (Linear Age Term) ^1^	Predicted Value (Fit) [95% CI]
Foveal bulge (prevalence)	Chronological	0.01	17–44 (*n* = 130): 0.93 [0.29, 1.0]
			45–59 (*n* = 100): 0.82 [0.12, 0.99]
			60–74 (*n* = 66):0.68 [0.05–0.99]
			75–92 (*n* = 12): 0.48 [0.02–0.98]
	Biological	<0.01	17–44 (*n* = 140): 0.92 [0.26, 1.0]
			45–59 (*n* = 94): 0.78 [0.09, 0.99]
			60–74 (*n* = 68): 0.60 [0.04–0.98]
			75–92 (*n* = 6): 0.38 [0.01–0.96]

^1^ Effect size: odds ratio (OR) per year of age with 95% CI. Note: Wide confidence intervals in older age groups reflect smaller subgroup sizes.

## Data Availability

The original contributions presented in this study are included in the article. Further inquiries can be directed to the corresponding author.

## Abbreviations

The following abbreviations are used in this manuscript:

OCT	Optical coherence tomography
EZ	Ellipsoid zone
CFT	Central foveal thickness
ILM	Internal limiting membrane
GAM	Generalized additive models
AIC	Akaike information criterion
COS	Cone outer segments
RPE	Retinal Pigment Epithelium
CRP	C-reactive Protein
ICC	Intraclass Correlation Coefficient
OR	Odds Ratio
SD	Standard Deviation

## Appendix A

**Table A1:** Reliability of Foveal Parameter Measurements (ICC).

Parameter	ICC	95% Confidence Interval
Central Foveal Thickness	0.988	0.975–0.994
Foveal Pit Depth	0.974	0.952–0.986
Foveal Pit Diameter	0.965	0.938–0.979
Mean Foveal Pit Slope	0.958	0.924–0.974

Note. All ICC values indicate excellent intergrader reliability.

**Table A2:** Sensitivity analysis of foveal pit edge definition using alternative derivative thresholds.

Derivative Threshold	Foveal Pit Depth (Microns), Mean ± SD	Mean Absolute Difference vs. 50% (Microns)	Pearson r vs. 50%
40% of max slope	118.6 ± 21.4	6.2	0.97
50% of max slope (reference)	120.1 ± 21.1	-	-
60% of max slope	117.9 ± 22.0	7.1	0.96

Sensitivity analysis was performed to assess the robustness of foveal pit edge definition based on the first derivative of the ILM profile. Measurements obtained using alternative thresholds (40% and 60% of the maximum slope) showed high agreement with the primary 50% threshold, as indicated by small mean absolute differences and strong correlations.

**Table A3:** Akaike information criterion for the tested models.

Feature	Age Type	AIC (Constant)	AIC (Linear)	AIC (Gam)
Foveal bulge	Chronological	426.4387	403.8320	best k = 5 410.8125
Foveal bulge	Biological	426.4387	396.3458	best k = 5 396.3666
Foveal thickness	Chronological	2790.3378	2791.8402	best k = 5 2792.1508
Foveal thickness	Biological	2790.3378	2791.9163	best k = 10 2792.2123
Foveal pit depth	Chronological	2610.3224	2610.9331	best k = 5 2611.0732
Foveal pit depth	Biological	2610.3224	2610.9672	best k = 5 2611.1081
Foveal pit diameter	Chronological	3929.3083	3929.7505	best k = 5 3930.0687
Foveal pit diameter	Biological	3929.3083	3929.7322	best k = 5 3931.6543
Foveal slope	Chronological	1117.3653	1118.2604	best k = 10 1118.6629
Foveal slope	Biological	1117.3653	1118.0126	best k = 5 1118.3328

**Table A4:** Fixed- and random-effect estimates of mixed-effects models for foveal structural parameters and foveal bulge prevalence.

Parameter	Type of Age	SD Patient [95% CI] ^1^	Residual SD [95% CI] ^2^	Effect Size (R^2^adj or OR [95% CI]) ^3^
Foveal thickness (microns)	Chronological	20.04 [17.33–23.18]	14.73 [13.17–16.47]	0.65 [0.62–0.68]
	Biological	20.04 [17.33–23.18]	14.73 [13.17–16.47]	0.65 [0.62–0.68]
Foveal pit depth (microns)	Chronological	20.62 [18.19–23.37]	9.77 [8.74–10.92]	0.82 [0.80–0.83]
	Biological	20.62 [18.19–23.37]	9.77 [8.74–10.92]	0.82 [0.80–0.83]
Foveal pit diameter (microns)	Chronological	262.25 [233.30–292.80]	77.11 [68.96–86.22]	0.92 [0.91–0.93]
	Biological	262.25 [233.30–292.80]	77.11 [68.96–86.22]	0.92 [0.91–0.93]
Foveal slope steepness (°)	Chronological	1.47 [1.28–1.69]	0.93 [0.83–1.04]	0.71 [0.69–0.74]
	Biological	1.47 [1.28–1.69]	0.93 [0.83–1.04]	0.71 [0.69–0.74]
Foveal bulge (prevalence) *	Chronological	2.29 [1.51–3.49]	-	0.949 [0.918–0.981]
	Biological	2.21 [1.43–3.40]	-	0.946 [0.916–0.976]

^1^ SD Patient—standard deviation of the patient-level random intercept (σ patient, same units as the outcome). ^2^ Residual SD—residual standard deviation (σ residual). ^3^ Effect size: R^2^adj for continuous parameters with 95% CI; odds ratio (OR) per year of age with 95% CI for foveal bulge. * Selected model for foveal bulge–logistic (linear age). For all other parameters–constant. Note: Table A4 presents full fixed- and random-effect estimates of the mixed-effects models for transparency and reproducibility. Predicted values and inferential statistics are reported in Table 3, Table 4 and Table 5.
